# Art Engagement and Risk of Type 2 Diabetes: Evidence From the English Longitudinal Study of Ageing

**DOI:** 10.3389/ijph.2023.1605556

**Published:** 2023-02-20

**Authors:** Xiaowen Wang, Jie Jiang, Yonghua Hu, Li-Qiang Qin, Yuantao Hao, Jia-Yi Dong

**Affiliations:** ^1^ Peking University Center for Public Health and Epidemic Preparedness & Response, Peking University, Beijing, China; ^2^ Public Health, Department of Social Medicine, Osaka University Graduate School of Medicine, Osaka, Japan; ^3^ Department of Epidemiology and Biostatistics, School of Public Health, Peking University Health Science Center, Beijing, China; ^4^ Department of Nutrition and Food Hygiene, School of Public Health, Soochow University, Suzhou, China

**Keywords:** cohort study, type 2 diabetes, prospective study, art engagement, English Longitudinal Study of Ageing

## Abstract

**Objectives:** To examine the prospective association between art engagement and the risk of type 2 diabetes.

**Methods:** Adults aged ≥50 from the English Longitudinal Study of Ageing were asked about the frequency of art engagement, including going to the cinema, the art gallery or museum, and the theatre, a concert, or the opera. Cox proportional hazards regression models were used to examine the risk of type 2 diabetes associated with art engagement.

**Results:** During a median follow-up of 12.2 years, we identified 350 cases of type 2 diabetes from 4,064 participants through interviews. After multivariable adjustment, compared with people who never went to the cinema, those going to the cinema frequently had a significantly lower risk of developing type 2 diabetes (HR = 0.61, 95% CI: 0.44–0.86). After further adjustment for socioeconomic factors, the association was slightly attenuated but remained statistically significant (HR = 0.65, 95% CI: 0.46–0.92). Similar results were found for going to the theatre, a concert, or the opera.

**Conclusion:** Frequent art engagement may be associated with a lower risk of type 2 diabetes, which was independent of individuals’ socioeconomic factors.

## Introduction

Art is a way to express feelings and emotions, exerting positive effects on one’s psychological states as well as physiological parameters. Participating in art activities is prone to be enjoyable, relaxing, active, stress-relieving, and socially interactive. The report of Taking Part showed that 77.5% of adults in England engaged in at least one art activity in 2013 and 2014 (1). There has been increasing interest in the role of art involved in health and wellbeing. A number of studies have found that art or cultural engagement was associated with a wide range of health outcomes, including wellbeing (2–4), chronic pain (5), anxiety (6), depression (7), dementia (8), cardiovascular diseases (9–11), and the chance of survival (12, 13).

The psychosomatic perspective on chronic diseases, especially cardiometabolic diseases, has received considerable attention. For instance, several previous cohort studies from Europe suggested that cultural or art participation could contribute to a lower risk of cardiovascular mortality (9, 10, 13). Our recent study among a Japanese population of 56,000 found that having hobbies, including engaging in enjoyable social activities (e.g., attending theater/art exhibitions, traveling), was associated with a 20% reduction in the risk of developing cardiovascular diseases within a 16-year follow-up (11). Apart from observational studies, intervention trials also reported that listening to music had a positive impact on aortic stiffness and wave reflections, which are markers of cardiovascular fitness (14). In addition, there was evidence that music therapy might improve glycemia among type 2 diabetes patients (15).

Despite the various health benefits associated with art engagement introduced above, it is largely unknown whether or not art engagement is associated with the risk of type 2 diabetes. Furthermore, people with a high level of socioeconomic status are more likely to participate in art activities, raising the concern that associations of art engagement with human health outcomes might be confounded by individuals’ socioeconomic status. Hereby, we aimed to examine the prospective association between art engagement and the risk of incident type 2 diabetes with considerations on possible confounding by socioeconomic factors.

## Methods

### Participants

The participants enrolled in our study were from the English Longitudinal Study of Ageing (ELSA), which is a nationally representative study among people aged 50 and over living in England. The design, sampling, and study procedure have been described before (16). Briefly, the first survey was from 2002 to 2003 (wave1), and repeated surveys were conducted biennially. The last survey was 2018–2019 (wave 9). In each wave, participants were interviewed by detailed questionnaires for their demographic characteristics, socioeconomic status, lifestyles, medication use, and history of diseases. Anthropometric and biological measures were also taken by trained medical staff. Ethical approval for the ELSA was obtained from the National Research Ethics Service. All the participants provided informed consent before this survey.

In this study, we treated wave 2 (2004–2005) as the baseline because body mass index (BMI) was not assessed at wave 1. The latest wave available for analysis was wave 9 (2018–2019). A total of 6,918 participants who had complete data on art engagement (going to the cinema; or going to an art gallery or museum; or going to the theatre, a concert, or the opera) at baseline and provided follow-up data on diabetes were included. Participants who had a history of diabetes, heart diseases, stroke or cancers, or had no measure of BMI, or had an extreme BMI (i.e., <14 or >40 kg/m^2^) at baseline were excluded. Finally, a total of 4,064 participants were included in the current analysis.

### Assessment of Art Engagement

Participants were asked about the frequency of art engagement by three questions: “How often respondent goes to the cinema,” “How often respondent goes to an art gallery or museum” and “How often respondent goes to the theatre, a concert or the opera.” Six options were provided for each question: “Twice a month or more,” “About once a month,” “Every few months,” “About once or twice a year,” “Less than once a year,” and “Never.” Due to the limited number of participants, we regrouped them into three categories: never, infrequent (about once or twice a year; less than once a year), and frequent (twice a month or more; about once a month; every few months).

### Covariates

The covariates included age, sex, ethnicity, marital status, smoking status, drinking status, level of physical activity, depressive symptoms measured by the Centre for Epidemiological Studies Depression scale, and self-reported doctoral diagnoses of hypertension and high cholesterol. Socioeconomic factors included household non-pension wealth (income), educational qualification, and occupation defined by the National Statistics Socioeconomic Classification. Height and weight were objectively measured during the nurse visit at wave 2, and BMI was calculated as weight (kg)/height (m)^2^.

### Assessment of Outcome

Incident type 2 diabetes cases were identified from wave 3 (2006–2007) to wave 9 (2018–2019). In each wave, participants were asked whether they had diabetes or a high blood sugar diagnosis newly reported since their last interview. Previous study has reported a high concordance between self-reported and clinically diagnosed diabetes in the ELSA study (17). Participants were censored at the wave when the outcome was newly reported, the wave when drop-out occurred, or the latest wave 9, whichever came first.

### Statistical Analyses

Differences in baseline characteristics were tested by ANOVA test for continuous variables or Chi-Square test for categorical variables with multiple comparison test correction. To examine the association between art engagement and incident type 2 diabetes risk, we estimated hazard ratios (HRs) and 95% confidence intervals (CIs) by using the Cox proportional hazards regression model. The proportional hazards assumption was evaluated by likelihood ratio test, and no violations were detected (*p* > 0.05 for all tests). Individuals who were never engaged with art were treated as the reference group throughout the analysis. Model 1 was only adjusted for age. Model 2 was further adjusted for sex, ethnicity (white/non-white), marital status (six categories), smoking status (yes/no), drinking status (eight categories), level of physical activity (five categories), BMI (quintiles), depression (yes/no), history of hypertension (yes/no), and history of high cholesterol (yes/no). Model 3 was further adjusted for socioeconomic factors, including household income (quintiles), educational qualifications (seven categories), and occupation (nine categories). We also conducted interaction analysis to examine whether sex could modify the association between art engagement and risk of type 2 diabetes by adding a cross-product term between art engagement and sex into the models. In addition, we conducted a sensitivity analysis to test the stability of the association by excluding cases identified during the first 2 years of follow-up. The sample weights were calculated by using a combination of the design and non-response weights that are available to the users with the ELSA data, therefore all analytical models were weighted to be representative of the English population. All analyses were performed using SAS version 9.4 (SAS Institute Inc.). *p* values <0.05 were considered statistically significant except where otherwise stated.

## Results

The baseline characteristics of participants according to art engagement were presented in Table 1. Compared to those never going to cinema, participants going to cinema frequently were more likely to be younger, to have a lower BMI, a higher socioeconomic status, and a healthier lifestyle and less likely to have high blood pressure or depressive symptoms. Similar patterns were found for going to an art gallery or museum, and going to the theatre, a concert, or the opera (Table 1).

**TABLE 1:** Baseline characteristics of participants according to art engagement (Beijing, China. 2023).

	Going cinema			Going art gallery or museum			Going the theatre, a concert or the opera		
	Never	Infrequent	Frequent	Never	Infrequent	Frequent	Never	Infrequent	Frequent
No. of participants	1340	1652	889	1302	1849	680	1134	1824	981
Age, years	67.5 (9.5)	63.1 (7.9)*	61.8 (7.5)*	66.5 (9.6)	63.3 (8.2)*	63.1 (7.8)*	66.5 (9.5)	63.7 (8.4)*	63.4 (8.1)*
BMI, kg/m^2^	27.9 (4.5)	27.2 (4.0)*	26.9 (3.8)*	28.0 (4.5)	27.1 (4.0)*	26.8 (4.0)*	27.9 (4.5)	27.4 (3.9)*	26.6 (4.0)*
Men, %	45.4	44.9	40.7	43.2	45.1	45.4	49.9	41.8*	41.6*
Married, %	69.7	73.6*	69.3	66.9	74.7*	70.3*	68.2	73.0*	71.4*
White, %	98.7	99.1	98.5	98.5	99.1	98.9	98.2	98.8	99.6*
Highly Educated, %	6.7	15.2*	26.8*	4.3	14.8*	37.1*	4.7	13.5*	29.2*
Income in top quintile, %	13.9	27.8*	35.0*	11.7	27.8*	42.0*	9.4	26.1*	39.3*
Professional occupation, %	21.7	37.7*	48.4*	18.5	37.4*	58.8*	17.6	36.1*	51.2*
High blood pressure, %	28.6	26.4	22.5*	26.9	26.4	23.9	28.2	26.3	24.2*
High cholesterol, %	15.9	16.5	15.0	16.2	16.6	14.3	14.5	17.0	15.9
Depressed, %	8.8	6.8	4.8*	9.8	5.9*	4.5*	10.7	5.7*	5.1*
Current smokers, %	21.9	12.2*	6.6*	21.9	11.3*	8.1*	24.5	11.8*	7.0*
Daily drinkers, %	15.3	20.0*	22.3*	14.8	19.7*	26.4*	15.0	17.3	26.4*
High physical activity, %	18.4	24.2*	27.7*	17.3	25.4*	28.2*	16.4	24.5*	28.4*

*p-value <0.025 compared to the reference group with multiple test correction.

All values are presented as the mean (standard deviation) or percentage.

As shown in Table 2, we identified 350 cases of type 2 diabetes among 4,064 participants during a median of 12.2 years of follow-up. In the age-adjusted model, compared with people who never went to the cinema, those going to the cinema frequently had a significant lower risk of developing type 2 diabetes (HR = 0.46, 95% CI: 0.33–0.64). After further adjustment for sex, BMI, ethnicity, marital status, smoking status, drinking status, level of physical activity, depression, history of hypertension, and history of high cholesterol, compared with people who never went to the cinema, those going to the cinema frequently had a significant lower risk of type 2 diabetes (HR = 0.61, 95% CI: 0.44–0.86). After further adjustment for socioeconomic factors, including education level, occupation, and household income, the association was slightly attenuated but remained statistically significant (HR = 0.65, 95% CI: 0.46–0.92). Similarly, in the age-adjusted model, compared with people who never went to the theatre, a concert, or the opera, those going frequently had a significant lower risk of developing type 2 diabetes (HR = 0.40, 95% CI: 0.30–0.55). After multivariable adjustment, those who engaged frequently still had a significant lower risk of type 2 diabetes (HR = 0.59, 95% CI: 0.43–0.82). Further adjustment for socioeconomic factors changed the association a little (HR = 0.61, 95% CI: 0.43–0.87). The association for going to an art gallery or museum was detected in the age-adjusted model (HR = 0.60, 95% CI: 0.43–0.83) but disappeared after multivariable adjustment (HR = 0.92, 95% CI: 0.65–1.30 and HR = 1.03, 95% CI: 0.71–1.49).

**TABLE 2:** Association between art engagement frequency and risk of type 2 diabetes (Beijing, China. 2023).

	Never	Infrequent	Frequent
Going cinema			
No. of Cases	142	142	53
No. of Participants	1375	1669	895
HR, model 1	1.00	0.70 (0.55, 0.88)	0.46 (0.33, 0.64)
HR, model 2	1.00	0.84 (0.66, 1.08)	0.61 (0.44, 0.86)
HR, model 3	1.00	0.90 (0.69, 1.17)	0.65 (0.46, 0.92)
Going art gallery or museum			
No. of Cases	140	147	51
No. of Participants	1326	1867	691
HR, model 1	1.00	0.65 (0.51, 0.82)	0.60 (0.43, 0.83)
HR, model 2	1.00	0.81 (0.63, 1.03)	0.92 (0.65, 1.30)
HR, model 3	1.00	0.90 (0.69, 1.16)	1.03 (0.71, 1.49)
Going the theatre, a concert or the opera			
No. of Cases	142	142	59
No. of Participants	1156	1847	992
HR, model 1	1.00	0.52 (0.41, 0.66)	0.40 (0.30, 0.55)
HR, model 2	1.00	0.61 (0.48, 0.78)	0.59 (0.43, 0.82)
HR, model 3	1.00	0.64 (0.49, 0.83)	0.61 (0.43, 0.87)

Model 1: age.

Model 2: further adjusted for sex, body mass index, marital status, ethnicity, smoking status, drinking status, level of physical activity, depressed, history of hypertension, and history of high cholesterol.

Model 3: further adjusted for education level, occupation, and income.

Interaction analysis showed that sex did not modify the association between art engagement and risk of type 2 diabetes (*p*-value for interaction = 0.29 for going to the cinema, 0.79 for going to an art gallery or museum, and 0.20 for going to the theatre, a concert, or the opera). We next performed a sensitivity analysis by excluding cases identified within the first 2 years of follow-up. The associations were stable and not materially altered.

## Discussion

In this longitudinal study, we observed that frequent art engagement, especially going to the cinema and going to the theatre, a concert, or the opera, was associated with a lower risk of developing type 2 diabetes. These findings were robust even after adjustment for socioeconomic factors, indicating the association of art engagement with risk of type 2 diabetes may be independent of individuals’ socioeconomic status.

To our knowledge, this is the first prospective cohort study to show a potential protective role of some forms of art engagement in the prevention of type 2 diabetes. A meta-analysis of five randomized controlled trials showed that art therapy, including music therapy and painting therapy, positively lower the level of blood glucose among people with diabetes (mean difference: −0.90, 95% CI: −1.03, −0.77; *p* < 0.0001), though there was no effect on glycated hemoglobin, indicating the potential improved function of hypothalamic-pituitary-adrenal (HPA) axis and reduction of cortisol level (18). In addition, in line with our study, several attempts have been made to explore some specific art activity or interventions associated with type 2 diabetes. For example, a clinical trial observed a significant difference in glucose levels before and after music therapy (*p* < 0.001), and listening to the music was associated with a 20% level of glucose reduction among people with type 2 diabetes (15). Another randomized controlled trial suggested that yoga and music therapy in combination with standard care exerted beneficial effects on glycemic control, lipid profile, weight, and self-efficacy in type 2 diabetes management (19). Art, music, and dance therapy has been categorized into mind-body medicine as complementary and alternative healthcare (20). Our study also indicated that collaboration between conventional diabetes preventing measures and art or relaxation engagement is recommended.

Potential mechanisms warrant discussion. Art engagement has been shown to be associated with good satisfaction with life (4), improved mental health, and lower levels of anxiety and depression (7, 21). The stress response was partly involved in the pathogenesis of type 2 diabetes. Psychological disorders may result in HPA abnormalities, high levels of cortisol, and neuroendocrine dysfunctions, leading to insulin resistance and abdominal obesity (15, 22). The use of biofeedback and relaxation was associated with chronic stress responding, peripheral vasoconstriction, muscle tension, and level of cortisol and catecholamines. It was reported that biofeedback-assisted relaxation therapy might lower blood glucose and glycated hemoglobin among people with type 2 diabetes (23). It is worth noting that art engagement in the form of art gallery/museum visits was not shown to have statistically significant associations with type 2 diabetes in the multivariable models. The perception of melody, harmony, and rhythm of sound and music, such as going to the cinema or going to the theatre, a concert, or the opera, might provide more enhanced neurophysiological benefits that build mental resilience and offset the detrimental physiological process implicated in type 2 diabetes, including the release of glucose and lipids into the circulation, poor glycemic control, neuroendocrine dysfunction, chronic inflammation, and increased blood pressure (24–26). It was suggested that classical music could alter the activity of the neurotransmitter epinephrine by which influencing the glucose release and β-cells function (15). Nevertheless, this finding should be also interpreted with caution due to residual confounders or limited statistical power. Further studies are helpful to evaluate the impact of specific type of art engagement on more detailed physiological and metabolic parameters and outcomes.

Additionally, outside art engagement may combine a number of protective factors associated with type 2 diabetes, including increasing physical activities (13), enhancing cognitive and perceptual functions (8, 27), reducing loneliness (28) and improving social connectedness (29, 30). Engagement with music or other media acts as a social surrogate that could be a replacement for interaction with others (31). Similarly, going to cinema, the theatre, or the opera enabled adults to connect with the world by increasing social engagement and communication. In addition, some other outdoor social activities (such as bingo, dancing, church service, traveling, volunteering, study circles/courses), were observed to decrease the risk of diabetes and its complications (32). Social networks or relationships are essential for type 2 diabetes self-management through sharing knowledge, behavior change, and access to healthcare resources (33, 34). On the contrary, home art activities such as TV viewing are inclined to be sedentary and introversive, showing higher risk of type 2 diabetes after adjusting for physical activity. Sedentary behaviors of home art engagement might further exert adverse effect on glucose metabolism in the large skeletal muscles involved in posture (35). In addition, individuals who were often engaged with outside art activities might be of high socioeconomic status. A previous study indicated that only part of the association was due to differences in socioeconomic status (e.g., 9.1% explained by wealth) among those with and without art engagement (13). Nevertheless, statistical adjustment for socioeconomic factors did not alter the association.

The present study was strengthened by using a nationally representative population and a prospective cohort design with a long duration of follow-up. Several limitations should be addressed. First, this study was conducted among the English population, generalizability to other populations should be cautioned. Second, we only assessed art engagement at one timepoint, changes in art engagement behavior during follow-up were very likely to have biased the association toward the null. However, art engagement serves as a hobby that could be relatively stable over time and reflect habitual behaviors (11). Third, the assessment of type 2 diabetes was according to self-reported physician diagnosis, which possibly resulted in misclassification. Nevertheless, a high concordance has been previously reported between self-reported and clinically diagnosed cases of diabetes in the ELSA study (17). Furthermore, though multiple established risk factors including socioeconomic factors were adjusted, residual confounding cannot be excluded due to the observational design, potential measurement errors, and other unmeasured factors (e.g., dietary intakes).

In summary, frequent art engagement may be associated with a lower risk of type 2 diabetes, which appeared to be independent of individuals’ socioeconomic status. Whether art engagement could contribute to diabetes prevention in the general population warrants further investigations.
